# Special Issue: Alternative Therapeutic Approaches of *Candida* Infections

**DOI:** 10.3390/jof8020170

**Published:** 2022-02-10

**Authors:** Renátó Kovács

**Affiliations:** 1 Department of Medical Microbiology, Faculty of Medicine, University of Debrecen, Negyerdei krt. 98, 4032 Debrecen, Hungary; kovacs.renato@med.unideb.hu; 2 Faculty of Pharmacy, University of Debrecen, Negyerdei krt. 98, 4032 Debrecen, Hungary

In recent decades, the prevalence of resistant fungal isolates has been steadily increasing both in veterinary and human medicine as well as in agriculture [1,2]. This global phenomenon can be explained the overuse of a limited number of antifungal drugs, global warming, and further anthropogenic effects. In addition, the combined effect of these factors has induced the emergence of previously rarely or never isolated fungal species such as multi- or panresistant *Candida auris*, azole-resistant *Aspergillus* species, or *Lomentospora prolificans* [1,2,3,4]. Nowadays, the lack of totally new antifungal agents further exacerbates the problem. Therefore, it is necessary to identify and introduce new antifungal drugs or alternative therapeutic approaches in clinical practice.

This Special Issue addresses current state-of-the-art investigations with regard to alternative therapeutic approaches against different potentially resistant *Candida* species. A total of nine papers were published in this Special Issue, including one review and eight original articles. The review article presents our current knowledge about *C. glabrata,* including its resistance, adaptation, and survival strategies [5]. Andrade et al. (2020) showed that certain *Copaifera* leaf extracts, especially *Copaifera paupera* and *Copaifera reticulata* leaf extracts, inhibited *C. glabrata* biofilm formation and increased the cell vacuolization and cell membrane damage of this species [6]. Regarding other plant-derived molecules, the Colombian essential oil *Ruta graveolens* showed high activity against clinically relevant *Candida* species; moreover, it demonstrated a remarkable synergizing effect in combination with traditional antifungal drugs [7]. In another study, the antifungal activity of the phenolic compounds ellagic acid and caffeic acid phenethyl ester was examined against *C. auris* isolates, where the caffeic acid phenethyl ester reduced the biomass and the metabolic activity of *C. auris* biofilm and decreased its adhesion to cultured human epithelial cells, indicating a potent antifungal and antivirulence effect [8]. It is noteworthy that one published paper focused on an animal-derived compound in terms of an antifungal effect. Kakar et al. (2021) demonstrated new perspectives in the antimicrobial activity of the amphibian temporin B. Both of the examined temporin B analogs inhibited the growth of planktonic and sessile cells of *Candida* species. In addition, they demonstrated favorable cytotoxicity properties as well [9]. Araujo et al. (2020) prepared a novel cetylpyridinium chloride nanocarrier using iron oxide nanoparticles conjugated with chitosan and assessed its antifungal and cytotoxic effects [10]. They observed a remarkable antifungal effect exerted by this complex against planktonic and sessile *Candida* cells, supporting the usage of this compound for the treatment of oral fungal infections [10]. Gómez-Casanova et al. (2021) evaluated the in vitro effect of the combination of cationic carbosilane dendrons derived from 4-phenylbutyric acid in the presence of AgNO_3_ and EDTA [11]. They found that the second generation dendron with AgNO_3_ or EDTA can decrease the viability of the biofilms formed by *C. albicans* [11]. A comprehensive study performed by Jakab et al. (2021) showed the negative effect of protein phosphatase Z1 deletion on oxidative stress tolerance, helping the development of further, novel antifungal drugs [12]. Furthermore, this phenotype can enhance the effect of clinically applied betamethasone combined with menadione [12]. Drug repurposing may be a potential alternative therapeutic approach in certain types of fungal infections [13]. Santos et al. (2021) examined the effects of lopinavir, an aspartic protease inhibitor in HIV therapy, on *C. albicans* by using in silico, in vitro, and in vivo approaches [14]. Based on their findings, lopinavir inhibits the yeast-to-hyphae transition, disturbs the ergosterol synthesis, blocks the adhesion to epithelial cells, and reduces the secreted aspartyl proteinase production by *Candida* cells [14].

In summary, this Special Issue is a great resource that highlights novel and innovative work on alternative therapeutic approaches. Hopefully, this Special Issue will be able to stimulate further exciting research in the future.
